# Org24598, a Selective Glycine Transporter 1 (GlyT1) Inhibitor, Reverses Object Recognition and Spatial Memory Impairments Following Binge-like Ethanol Exposure in Rats

**DOI:** 10.3390/molecules29246017

**Published:** 2024-12-20

**Authors:** Joanna Filarowska-Jurko, Pawel Grochecki, Ewa Gibuła-Tarlowska, Joanna Listos, Ewa Kedzierska, Justyna Socha, Irena Smaga, Tymoteusz Slowik, Małgorzata Filip, Jolanta H. Kotlinska

**Affiliations:** 1 Department of Pharmacology and Pharmacodynamics, Medical University of Lublin, Chodzki 4a, 20-093 Lublin, Poland; joanna.filarowska@gmail.com (J.F.-J.); pawel.grochecki@umlub.pl (P.G.); ewa.gibula-tarlowska@umlub.pl (E.G.-T.); joanna.listos@umlub.pl (J.L.); ewa.kedzierska@umlub.pl (E.K.); justynasocha97@gmail.com (J.S.); 2 Department of Drug Addiction Pharmacology, Maj Institute of Pharmacology Polish Academy of Sciences, Smetna 12, 31-343 Krakow, Poland; smaga@if-pan.krakow.pl (I.S.); mal.fil@if-pan.krakow.pl (M.F.); 3 Experimental Medicine Center, Medical University, Jaczewskiego 8, 20-090 Lublin, Poland; tymoteusz.slowik@umlub.pl

**Keywords:** ethanol, withdrawal, memory, Org24598, novel object recognition, Barnes-maze task

## Abstract

The N-methyl-D-aspartate (NMDA) glutamate receptor is a major target of ethanol, and it is implicated in learning and memory formation, and other cognitive functions. Glycine acts as a co-agonist for this receptor. We examined whether Org24598, a selective inhibitor of glycine transporter1 (GlyT1), affects ethanol withdrawal-induced deficits in recognition memory (Novel Object Recognition (NOR) task) and spatial memory (Barnes Maze (BM) task) in rats, and whether the NMDA receptor glycine site participates in this phenomenon. Male Wistar rats were habituated to NOR or BM tasks, and then received binge-like intragastric ethanol administration (5 days, 5 g/kg). After ethanol withdrawal, Org24598 (0.1, 0.3, and 0.6 mg/kg) was administered 30 min before NOR (day 10 of withdrawal) or the reversal learning phase of BM (day 11–13 of withdrawal) task. The expression of GluN1 and GluN2B subunits of NMDA receptors were measured in the perirhinal cortex (PRC) and hippocampus (HIP) after termination of NOR. In the BM task, a glycine antagonist, L-701,324 (5 mg/kg), was administered 30 min before Org24598 to confirm the involvement of the NMDA receptor glycine site in the effects of Org24598. Our study showed that binge-like ethanol administration induced recognition and spatial memory impairments after withdrawal in rats. Additionally, an up-regulation of GluN1 and GluN2B subunits of the NMDA receptor was observed in the HIP and PRC on day 11 of abstinence. Org24598 ameliorated memory loss and normalized the expression of these subunits. L-701,324 reversed the effect of Org24598. Thus, NMDA receptor glycine sites are important in ethanol withdrawal-induced memory impairments.

## 1. Introduction

Alcohol consumption in humans leads to impairments in decision-making, problem-solving, as well as learning and memory abilities [1,2]. The N-methyl-D-aspartate (NMDA) receptor is crucial for brain development, synaptic plasticity, and cognitive functions like learning and memory [3]. This receptor is also recognized as a primary target of ethanol in the brain, contributing to several ethanol-related effects, such as ethanol-induced excitotoxicity and disruptions in synaptic plasticity [4,5].

The NMDA receptor is an ionotropic glutamate receptor that binds glutamate with high affinity, uses glycine or D-serine as co-agonists, and is voltage-dependently blocked by Mg^2+^. When open, it allows sodium and calcium ions to enter the neuron, causing depolarization [6,7]. It consists of two GluN1 subunits, essential for glycine binding, and two GluN2 subunits, which bind glutamate [5]. Ethanol acts as an allosteric inhibitor of the NMDA receptor, reducing the frequency and duration of channel opening [4,5].

Another brain target of ethanol is the inhibitory glycine receptor. Animal models have shown that the glycine receptor in the nucleus accumbens (nAc) serves as a key entry point for ethanol’s effects on the mesolimbic dopamine system [8,9]. Additionally, in vivo studies found that activating glycine receptors in the nAc, either through a receptor (agonist) or a glycine transporter1 (GlyT1) inhibitor, increases dopamine release and reduces ethanol consumption. In contrast, applying strychnine, a selective glycine receptor antagonist, decreases dopamine levels and increases ethanol intake [10]. Therefore, glycine plays two essential roles as a neurotransmitter: acting as an agonist at inhibitory glycine receptors (strychnine-sensitive glycine receptors) and a co-agonist at excitatory NMDA receptors (strychnine-insensitive glycine sites) [11,12].

Glycine levels in the extracellular space are controlled by glycine transporters (GlyT), which include two subtypes: GlyT1 and GlyT2 [12]. GlyT2 is predominantly expressed presynaptically on glycinergic neurons located mainly in the caudal regions of the central nervous system (CNS), where it plays a key role in replenishing the presynaptic glycine pool for synaptic vesicles [13]. In contrast, GlyT1 is primarily found on glial cells and is widely distributed in the CNS, where it terminates postsynaptic activity at inhibitory glycine receptors and prevents the glycine-binding sites on NMDA receptors from becoming saturated [14]. Molecular studies have indicated that GlyT1 physically associates with postsynaptic density protein 95 (PSD95), a protein associated with NMDA receptors, suggesting that GlyT1 is localized near these receptors. This proximity enables GlyT1 to effectively modulate the activity of NMDA receptors [15].

Inhibition of GlyT1 leads to an increase in synaptic glycine levels in the CNS [14,16]. Blocking GlyT1 enhances synaptic activity and promotes long-term potentiation (LTP), which is consistent with the role of NMDA receptors in synaptic plasticity [17]. Studies in rodents have demonstrated that various GlyT1 inhibitors can counteract MK-801 (a noncompetitive NMDA receptor antagonist)-induced impairments in LTP [18] and recognition memory [19].

Org24598 is a highly selective sarcosine-based inhibitor of GlyT1, with minimal effects on GlyT2 and other transporters and receptors [20]. Previous research suggests that Org24598 is the most effective glycinergic agent for reducing alcohol consumption when compared to direct glycine receptor agonists, likely because it enhances glycine receptor neurotransmission [13,21,22,23,24,25,26]. However, Lidö et al. [27] demonstrated that the inhibitory effect of GlyT1 inhibitors (e.g., Org25935) on ethanol consumption [27,28] is not attenuated by L-701,324, an NMDA receptor glycine site antagonist, but is attenuated by strychnine, a glycine receptor antagonist.

It has been established that the GlyT1 inhibitor SSR504734 enhances working memory in mice [29] and that GlyT1 inhibitors mitigate cognitive symptoms induced by NMDA receptor antagonists [30,31,32]. Our previous research demonstrates that Org24598 improves cognitive functions, such as cognitive flexibility (reversal learning), in ethanol-exposed, maternally separated rats. This effect was reversed by L-701,324, indicating the involvement of NMDA receptor glycine sites in these cognitive processes [26].

In the present study, we investigated whether Org24598, a GlyT1 inhibitor, could attenuate deficits in recognition memory and spatial memory flexibility during withdrawal from binge-like ethanol exposure in adult male rats. Considering previous evidence that Org24598’s effects may involve glycine signaling in specific brain regions, we also examined its influence on ethanol-induced changes in NMDA receptor subunits (GluN1 and GluN2A-B) in memory-relevant structures, including the perirhinal cortex (PRC) and hippocampus (HIP). These regions are critical for distinct memory processes: the PRC for object recognition memory and the HIP for spatial and episodic memory [33]. Additionally, we evaluated whether L-701,324 could reverse the effects of Org24598 in the reversal learning task. This approach allowed us to assess the interplay between GlyT1 inhibition and NMDA receptor modulation in mitigating ethanol-induced cognitive deficits.

## 2. Results

### 2.1. NOR Task

**Figure 1:**
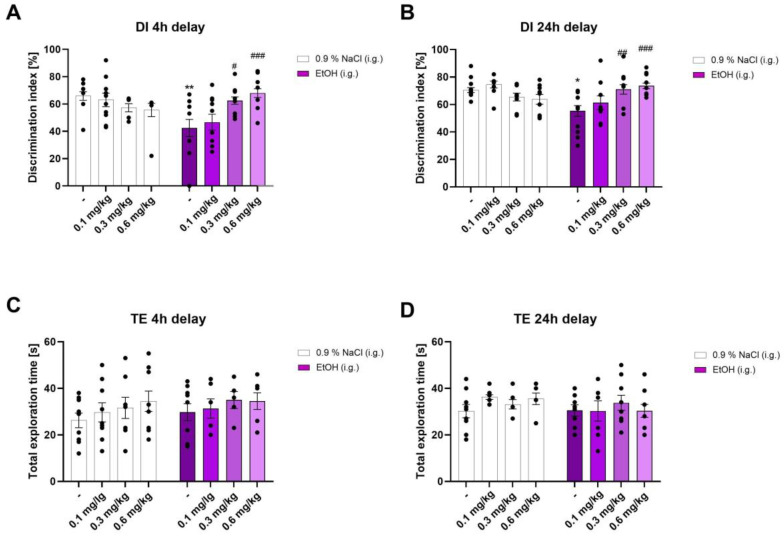
The influence of Org24598 on recognition memory deficits (expressed as a discrimination index) induced by binge-like ethanol administration in rats, measured on the 10th day of ethanol withdrawal at (**A**) 4 h and (**B**) 24 h after Org24598 administration in the NOR task. Total exploration time displayed by different groups of rats at (**C**) 4 h and (**D**) 24 h after Org24598 administration in the NOR task. Each column represents the mean ± SEM. * *p* < 0.05; ** *p* < 0.01 vs. 0.9% NaCl + Vehicle; ^###^ *p* < 0.001; ^##^ *p* < 0.01; ^#^ *p* < 0.05 vs. EtOH (i.g.) + Vehicle. Org—Org24598; EtOH—ethanol.

On the tenth day of ethanol abstinence, during the testing session (T2B) (short-term memory), two-way ANOVA did not indicate a statistically significant effect of pre-treatment (ethanol) [F (1, 87) = 2.883; *p* > 0.05], nor treatment (Org24598) [F (3, 87) = 1.390; *p* < 0.05]; however, two-way ANOVA revealed a significant effect of pre-treatment × treatment interaction [F (3, 87) = 7.285, *p* < 0.001]. Post hoc (Bonferroni) test showed that the ethanol withdrawal rats discriminated worse against the novel object than the saline-treated group (*p* < 0.01). Moreover, a single injection of Org24598 at the dose of 0.3 (*p* < 0.05) and 0.6 (*p* < 0.01), but not at the dose of 0.1 (*p* > 0.05) administered 30 min before the testing session (T1B), significantly increased the DI in ethanol-treated animals. Also, analysis of the total exploration times during T1B did not show a statistically significant influence of pre-treatment [F (1, 55) = 0.5601; *p* > 0.05], treatment [F (3, 55) = 1.124; *p* > 0.05] or pre-treatment × treatment interaction [F (3, 55) = 0.08708] (Figure 1).

After a 24 h delay of ethanol abstinence, during the testing session (T2C) (at a retention interval of 24 h), two-way ANOVA did not indicate a statistically significant effect of pre-treatment (ethanol) [F (1, 88) = 1.891; *p* > 0.05] nor a significant effect of treatment (Org24598) [F (3, 88) = 1.528; *p* > 0.05]. However, two-way ANOVA revealed a significant effect of pre-treatment × treatment interaction [F (3, 88) = 7.675; *p* < 0.001]. Post hoc (Bonferroni) test showed that the ethanol withdrawal rats discriminated worse against the novel object than the saline-treated group (*p* < 0.05). Moreover, a single injection of Org24598 at the dose of 0.3 (*p* < 0.01) and 0.6 (*p* < 0.001), but not 0.1 (*p* > 0.05), 30 min before the first testing session (T2B) significantly increased the DI in the ethanol-treated animals (*p* < 0.01). Also, analysis of the total exploration time during T2B did not show a statistically significant influence of pre-treatment [F (1, 57) = 1.664, *p* > 0.05], treatment [F (3, 57) = 0.5473; *p* > 0.05], nor pre-treatment × treatment interaction [F (3, 57) = 0.7724; *p* > 0.05] (Figure 1).

### 2.2. BM Task

**Figure 2:**
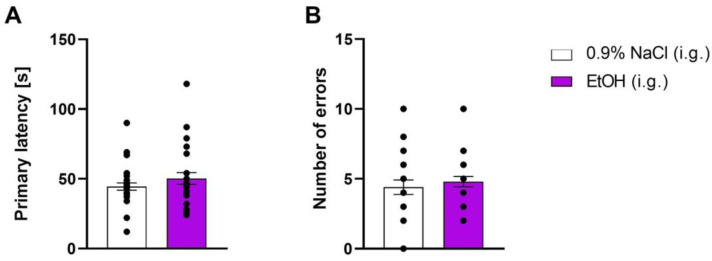
The influence of ethanol binge-like administration on spatial memory retention measured as (**A**) primary latency and (**B**) number errors measured on the 11th day of ethanol withdrawal in the BM task. Each column represents the mean ± SEM. EtOH—ethanol.

Ten days after ethanol withdrawal, during the probe trial, Student’s *t*-test did not show any significant effect of ethanol administration on spatial memory, measured by primary latency (t = 1.173, df = 58; *p* > 0.05) (Figure 2A) and the number of errors (t = 0.6306, df = 58; *p* > 0.05) (Figure 2B).

**Figure 3:**
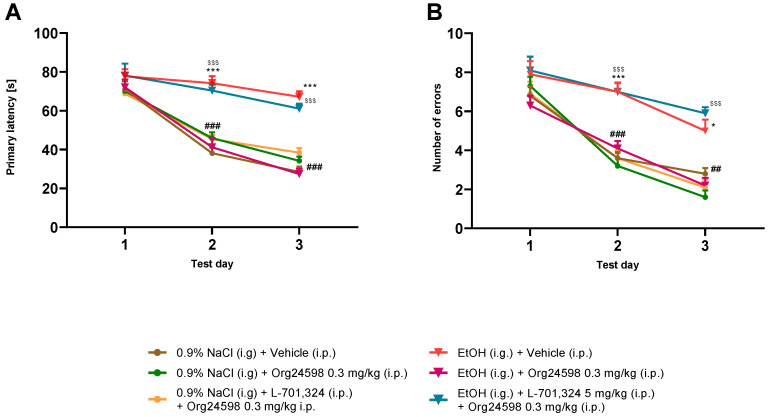
The influence of Org24598 on spatial memory flexibility measured as (**A**) primary latency and (**B**) number of errors during reversal learning measured on the 11th–13th day of ethanol withdrawal in the BM task. Impact of L-701,324 on the effect of Org24598 administered before each session of the reversal learning. Data are shown as mean ± SEM. * *p* < 0.05; *** *p* < 0.001 0.9% NaCl (i.g.) + Vehicle (i.p.) vs. EtOH (i.g.) + Vehicle (i.p.); ^##^ *p* < 0,01; ^###^ *p* < 0.001 EtOH (i.g.) + Vehicle (i.p.) vs. EtOH (i.g.) + Org24598 0.3 mg/kg (i.p); ^$$$^ *p* < 0.001 EtOH (i.g.) + L-701.324 (i.p.) + Org24598 0.3 mg/kg (i.p.). EtOH—ethanol.

In the reversal learning phase of the BM task, the results showed significant differences between tested groups. Two-way ANOVA of primary latency showed a significant effect of treatment [F (5, 54) = 27.24; *p* < 0.001], day of test [F (2, 108) = 119.5; *p* < 0.001], and interaction on these factors [F (10, 108) = 4.816]. Bonferroni’s multiple comparisons test showed significant differences between the 0.9%NaCl/Vehicle vs. the ethanol/Vehicle at the 2nd (*p* < 0.001) and 3rd day (*p* < 0.001), between the ethanol/Vehicle vs. the ethanol/Org24598 at the 2nd (*p* < 0.001) and 3rd day (*p* < 0.001) and between the ethanol/Org24598 vs. the ethanol/Org24598 + L-701,324 at the 2nd (*p* < 0.01) and 3rd day (*p* < 0.001) (Figure 3A).

Two-way ANOVA of the number of errors revealed a significant effect of treatment (F (5, 54) = 17.75; *p* < 0.001), day of test [F (2, 108) = 118.8; *p* < 0.001], and interaction of factors [F (10, 108) = 2.874; *p* < 0.01]. Bonferroni’s multiple comparisons test showed significant differences between the 0.9% NaCl/Vehicle vs. the ethanol/Vehicle at the 2nd (*p* < 0.001) and 3rd (*p* < 0.05) day, between the ethanol/Vehicle vs. the ethanol/Org24598 at the 2nd (*p* < 0.01) and 3rd (*p* < 0.05) day, and between the ethanol/Org24598 vs. the ethanol/Org24598 + L-701,324 at the 2nd (*p* < 0.01) and 3rd (*p* < 0.01) day (Figure 3B).

### 2.3. EPM

**Figure 4:**
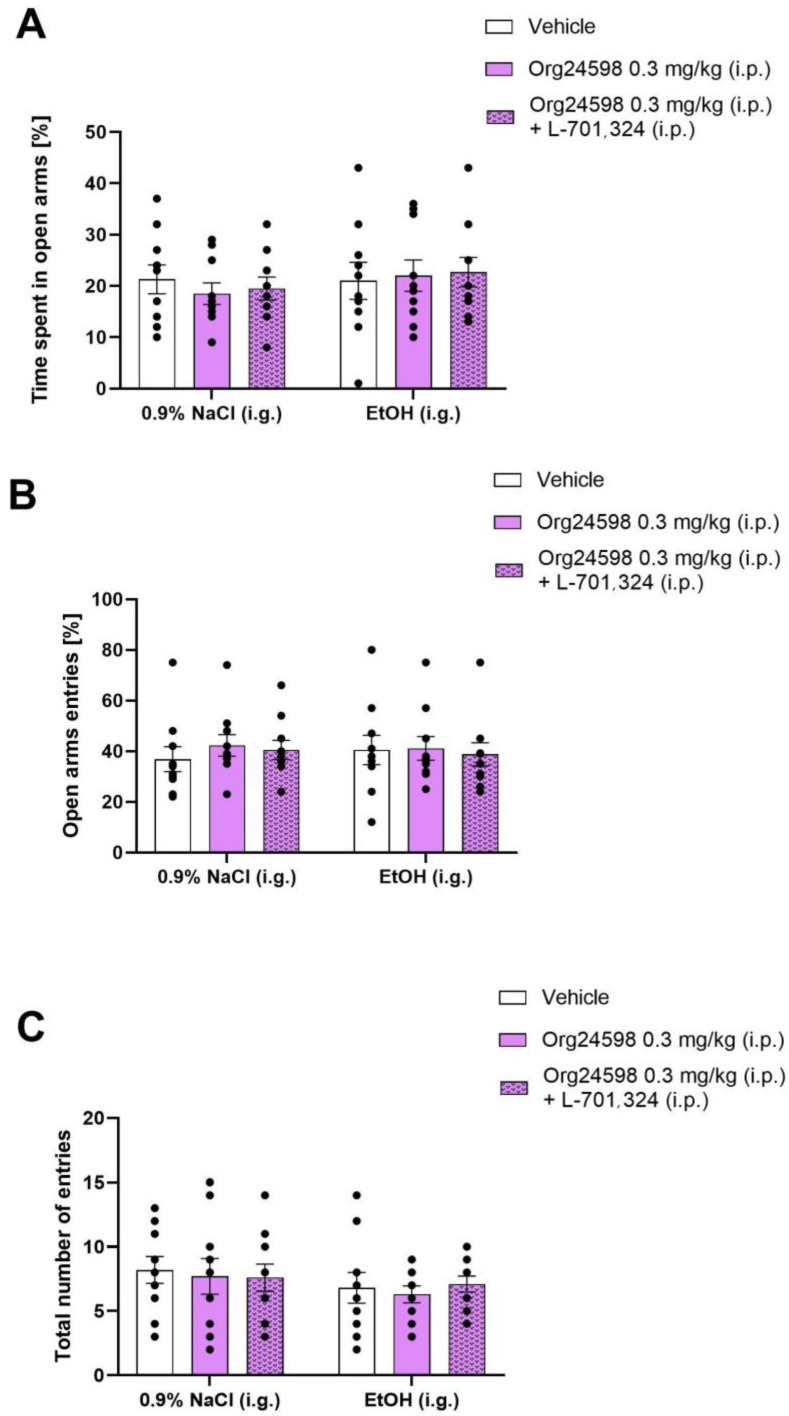
The influence of Org24598 on anxiety-like behavior in the EPM measured as (**A**) time spent in open arms, (**B**) open arms entries, and (**C**) total number of entries on the 11th day of ethanol withdrawal. Impact of L-701,324 on the effect of Org24598. Data are shown as mean ± SEM. EtOH—ethanol.

A two-way ANOVA revealed no statistically significant effect of ethanol [F (1, 54) = 0.8539; *p* > 0.05], Org24598/L-701,324 [F (2, 54) = 0.064; *p* > 0.05], or their interaction [F (2, 54) = 0.2791; *p* > 0.05] on the time spent in the open arms of the EPM (Figure 4A). Similarly, there was no significant effect of ethanol [F (1, 54) = 0.00368; *p* > 0.05], Org24598/L-701,324 [F (2, 54) = 0.2118; *p* > 0.05], or their interaction [F (2, 54) = 0.1929; *p* > 0.05] on the number of entries into the open arms (Figure 4B). Locomotor activity, measured by the total number of entries into both closed and open arms, was also unaffected by ethanol [F (1, 54) = 1.705; *p* > 0.05], Org24598/L-701,324 [F (2, 54) = 0.1237; *p* > 0.05], or their interaction [F (2, 54) = 0.1268; *p* > 0.05] (Figure 4C).

### 2.4. Perirhinal Cortex

**Figure 5:**
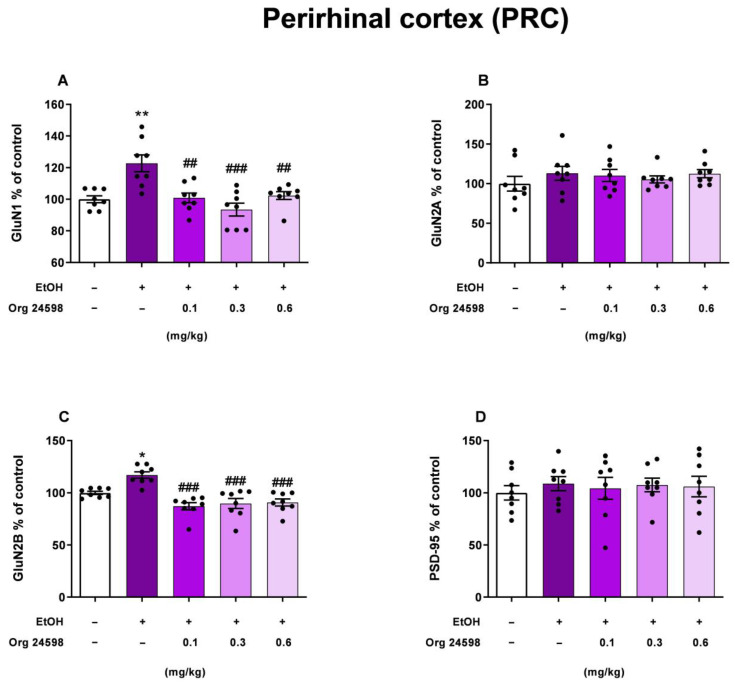
The influence of withdrawal from binge-like ethanol administration and Org24598 on the (**A**) GluN1; (**B**) GluN2A; (**C**) GluN2B; and (**D**) PSD-95 levels in the synaptosomal membranes of the PRC. Data are shown as mean ± SEM. * *p* < 0.05; ** *p* < 0.01 vs. 0.9% NaCl (i.g.) + Vehicle (i.p.); ^##^ *p* < 0.01; ^###^ *p* < 0.001 vs. EtOH (i.g.) + Vehicle (i.p.). EtOH—ethanol.

One-way ANOVA indicated a statistically significant effect of ethanol and Org24598 administration on the GluN1 receptor subunit protein level in the perirhinal cortex, in the ethanol-treated rats [F (4, 35) = 9.413; *p* < 0.001]. Post hoc analysis showed that ethanol withdrawal increased the GluN1 protein level (*p* < 0.01). What is more, Org24598 administration at the doses of 0.1 (*p* < 0.01), 0.3 (*p* < 0.001), and 0.6 mg/kg (*p* < 0.05) on the first day of ethanol withdrawal, 30 min prior to the testing trial, decreased GluN1 receptor protein expression in the ethanol-treated rats. Similarly, ethanol and Org24598 administration changed the GluN2B protein level during ethanol withdrawal [F (4, 35) = 12.91; *p* < 0.001]. Our results show that ethanol withdrawal increased GluN2B protein level (*p* < 0.05), but Org24598 at the doses 0.1, 0.3, and 0.6 administration reversed the effect of ethanol (*p* < 0.001). However, ethanol and Org24598 did not have an impact on the GluN2A [F (4, 35) = 0.5728; *p* > 0.05] and the PSD-95 [F (4, 35) = 0.730; *p* > 0.05] protein level in ethanol withdrawal rats (Figure 5).

### 2.5. Hippocampus

**Figure 6:**
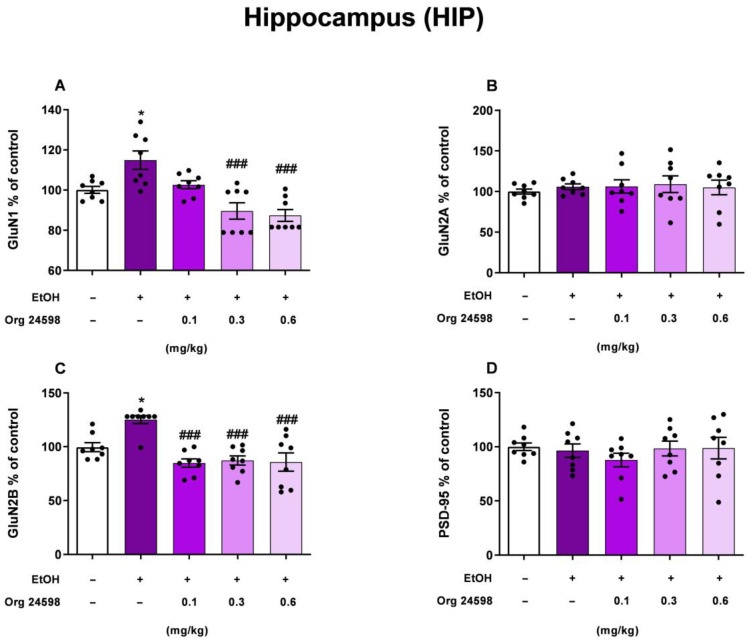
The influence of withdrawal from binge-like ethanol administration and Org24598 on the (**A**) GluN1; (**B**) GluN2A; (**C**) GluN2B; and (**D**) PSD-95 levels in the synaptosomal membranes of the HIP. Data are shown as mean ± SEM. * *p* < 0.05 vs. 0.9% NaCl (i.g.) + Vehicle (i.p.); ^###^ *p* < 0.001 vs. EtOH (i.g.) + Vehicle (i.p.). EtOH—ethanol.

One-way ANOVA indicated the statistically significant effect of ethanol and Org24598 administration on the NR1 receptor subunit protein level in the ethanol withdrawal rats [F (4, 35) = 11.65; *p* < 0.001]. Post hoc analysis showed that ethanol withdrawal increased the GluN1 protein level (*p* < 0.05). What is more, Org24598 administration at the doses of 0.3 (*p* < 0.001) and 0.6 mg/kg (*p* < 0.001) on the second day of ethanol withdrawal, 30 min prior to the testing trial, reversed the effect of ethanol. Similarly to the GluN1 subunit, ethanol and Org24598 administration changed the GluN2B protein level (F (4, 35) = 10.76; *p* < 0.001). Our results show that ethanol withdrawal increased GluN2B protein level (*p* < 0.05), but Org24598 administration decreased the effect of ethanol on GluN2B protein level at the doses of 0.1, 0.3, and 0.6 mg/kg (*p* < 0.001). However, treatment does not have an impact on the GluN2A (F (4, 35) = 0.1966; *p* > 0.05) and PSD-95 (F (4, 35) = 0.1966; *p* > 0.05) protein levels in the ethanol withdrawal rats (Figure 6).

## 3. Discussion

The study found that discontinuation of binge-like ethanol-induced impairment of recognition memory in the NOR task in rats. Org24598, a GlyT1 inhibitor, reduced recognition memory impaired by ethanol withdrawal, with a significant impact on long-term memory. In addition, at these doses, it did not affect locomotor activity or rat behavior in the EPM test. Moreover, our study demonstrated that the expression of the NMDA receptor subunits GluN1 and GluN2B in the PRC and HIP was increased after 11 days of abstinence and that Org24598 normalized these effects. Ethanol withdrawal did not affect spatial memory retrieval in the MB task but it did impair memory flexibility during reversal learning. Org24598 improved memory flexibility, and this effect was reversed by L-701,324, the NMDA receptor glycine site antagonist. Thus, our results suggest that the NMDA receptor glycine-binding site is involved in ethanol withdrawal-induced memory deficits in rats.

### 3.1. Effect of Org24598 on Memory Impairment Induced by Withdrawal of Binge-like Ethanol Administration in NOR Task

Excessive ethanol use leads to neurodegeneration in several brain structures, including the PRC and the HIP—the brain structures associated with cognitive deficits, including recognition and spatial memory [34]. Our previous study indicated that after the first 1–2 days (24–48 h) of withdrawal from repeated ethanol treatment (2.0 g/kg, e.g., for 7 days, once daily), the rats showed significant recognition memory deficits. Unlike the controls, rats treated with ethanol failed to recognize a novel object during the testing sessions [35]. The present study confirmed these data and indicated that the rats (familiarized with the NOR task environment before ethanol administration) showed a significant deficit in recognition (declarative) memory 10 and 11 days after ethanol withdrawal. In contrast to the control group, the animals that received ethanol did not recognize the new object during the test session. However, the methodology used in the present and the former experiment does not allow for a clear determination of whether the observed deficits are the result of learning or memory retrieval deficits. The observed deficits were evident in the absence of symptoms of abstinence, such as anxiety or impaired locomotor activity.

Animal models using continuous gastric infusion of ethanol differ significantly from the patterns of alcohol consumption in humans. However, such models allow for the precise control of nutritional status and enable the administration of high levels of ethanol, making them valuable tools for investigating how alcohol consumption leads to learning and memory impairments. In our study, we administered high doses of ethanol to achieve blood alcohol concentrations comparable to those observed in individuals with heavy alcohol consumption [36]. Importantly, these doses did not adversely affect the general health or survival of the animals, as all of them remained healthy throughout the study. Behavioral experiments commenced on the tenth day after ethanol withdrawal. However, other authors showed that object recognition ability was recovered after 10 weeks of withdrawal from 4-day binge ethanol treatment in rats [34]. Thus, it is possible that recognition memory is only temporarily affected by ethanol. The precise mechanism of cognitive impairment after withdrawal is not fully understood, and multiple mechanisms are likely to be involved [37].

Our results showed that ethanol-induced deficits in the NOR task were associated with an increase in the expression (“up-regulation”) of GluN1 (which contributes to the glycine binding site) and NR2B (which possesses the glutamate binding site) subunits after 11 days of abstinence, in brain structures, such as the PRC and HIP, in the absence of changes in the expression of the GluN2A subunit and PSD-95 protein in these structures. Studies by other authors indicate that “up-regulation” of the GluN2B subunit may be indicative of cognitive impairment [38]. Org24598 administration reversed the effects of ethanol in the PRC and HIP, although the effect was not dose-dependent. It has previously been shown that increasing the amount of glycine in the synaptic gap causes endocytosis and consequent internalization of the NMDA receptor [17,28], which in animals with “up-regulation” of NMDA receptors (for example, during a period of ethanol abstinence) can lead to normalization of its levels in the synaptic membrane. In fact, our results confirm the reduction (normalization) of GluN1 and GluN2B subunit expression to the level of control animals after Org24598 administration. This effect appears to have been transient, as PSD-95 protein [39] levels did not change. Thus, it can be speculated that Org24598 activates neuroprotective mechanisms against neurotoxicity induced by increased glutamate release during ethanol abstinence.

In our study, we observed alterations in the expression of GluN1 and GluN2B subunits in the PRC and HIP. These findings indicate that changes in NMDA receptor subunits occur in brain regions related to learning and memory. This supports our previous research suggesting that the HIP, alongside the PRC, is involved in ethanol-induced impairments in cognitive memory retrieval during ethanol withdrawal [40]. Additionally, our results confirmed a functional link between the GluN1 and GluN2B subunits in the PRC and HIP in processes related to declarative memory.

Chronic ethanol administration leads to an increase in the number and sensitivity of NMDA receptors in structures involved in memory processes [41,42,43,44]. Dysregulation may lead to impaired LTP [45]. It can be hypothesized that the action of Org24598 through indirect activation of NMDA receptors is related to the enhancement of LTP processes in brain structures that are responsible for declarative memory. The data obtained confirm that enhancement of NMDA receptor activity by increasing the glycine concentration improves cognitive function. We also cannot exclude the involvement of strychnine-sensitive glycine receptors in Org24598-induced memory enhancement, since, as indicated by literature data, glycine receptors, especially in the HIP, can assist NMDA receptors in enhancing their function in cognitive processes, especially in pathological states [46].

### 3.2. Effect of Org24598 on Memory Impairment Induced by Withdrawal of Binge-like Ethanol Administration in the BM Task

The negative effects of ethanol on spatial memory are due to damage in the function of the HIP—a brain structure involved in spatial memory processes [47]. Withdrawal from chronic ethanol causes a decrease in the number of pyramidal cells, especially in the CA1 and CA3 fields of the HIP, which likely contributes to the impairment of spatial memory. Our study showed that ethanol withdrawal had no effect on the recovery of previously learned memory. These animals found shelter on the day of the test properly without any problems. However, they had trouble learning the new task of finding a new shelter location.

The reversal learning test is used in experimental pharmacology to assess memory flexibility. Abnormalities in this test are associated with perseveration, or the persistent repetition of previously learned postural patterns. Published data indicate that both single and chronic ethanol administration damage memory flexibility [48,49]. Our study confirmed these reports.

Ethanol-induced memory impairment is associated with its effects on the glutamatergic system, particularly on NMDA receptors [50,51] in brain structures associated with memory processes. Thus, it appears that damage to memory flexibility may be due to dysfunction of NMDA receptors, which are particularly important in the formation of spatial memory [52]. Previous data have shown that blocking NMDA receptors prevents animals from learning a new shelter location, while the acquired memory is not impaired [53], which is consistent with our results. Animals that learned to find shelter prior to ethanol exposure showed no cognitive impairment in the test proper. The cognitive disturbances in these animals occurred during reversed learning. These may be due to the fact that NMDA receptor-dependent long-term depression (LTD) is required for behavioral flexibility and may act by weakening previously encoded memory traces when new information is learned [54]. This process can be disturbed in the ethanol withdrawal rats.

Numerous data from the literature indicate that repeated ethanol administration alters the activity of NMDA receptor subunits, including the GluN1 subunit, which is sensitive to glycine [55,56,57]. Moreover, our previous [40] and current studies showed “up-regulation” of the GluN1 subunit in rats after 10/11 days of ethanol abstinence. Published data suggest that these changes are dependent on the time, manner (continuous/intermittent), and dose of ethanol administered [58,59,60,61], and return to the control value within a few days of abstinence. In our study, the reverse learning test was conducted after 11–13 days of ethanol abstinence, so according to the results of other authors [62] and our data, the level of the GluN1 subunit may still have been changed during this time.

Furthermore, our study showed that administration of the GlyT1 inhibitor, Org24598, at a dose of 0.3 mg/kg for three consecutive days, starting on the 11th day of abstinence, improved reversal learning and memory plasticity. The animals learned the new location of the shelter faster and made fewer mistakes in the process than animals that received the vehicle instead of the Org24598 injection. Org24598, by modulating glycine levels in the synaptic cleft, can indirectly influence NMDA receptor function by altering co-agonist availability. Moreover, this effect was inhibited by the administration of L-701,324, an antagonist of the glycine site at the NMDA receptor, suggesting the involvement of the glycine site of the NMDA receptor in the pro-cognitive effects of Org24598. These data suggest that treatment with a GlyT1 inhibitor, Org24598, can ameliorate deficits in both LTP/LTD and learning that occur in a rat model of ethanol withdrawal after binge drinking.

In conclusion, this study demonstrates that binge-like ethanol exposure induces significant cognitive deficits in recognition and spatial memory in rats that are evident during withdrawal. The observed impairments correlated with increased expression of the GluN1 and GluN2B subunits of the NMDA receptor in key brain regions, namely the HIP and PRC. Treatment with Org24598, a selective GlyT1 inhibitor, ameliorated these deficits, likely through normalization of NMDA receptor subunit levels. The involvement of the NMDA receptor glycine site in these effects was confirmed by the reversal of Org24598’s action with the antagonist L-701,324. These findings underscore the therapeutic potential of GlyT1 inhibitors in addressing cognitive impairments associated with ethanol withdrawal, offering promising directions for further research into addiction-related cognitive deficits.

This research highlights the importance of understanding molecular pathways in ethanol-induced neurotoxicity and the potential for GlyT1 inhibitors to modulate these processes. By targeting specific mechanisms, such as the glycine site of the NMDA receptor, these findings provide a foundation for the development of novel therapeutic strategies aimed at mitigating cognitive dysfunction caused by ethanol use.

## 4. Materials and Methods

### 4.1. Animals

The study received approval from the Local Ethics Committee (125/2018) in Lublin. It was conducted in line with the National Institute of Health Guidelines for the Care and Use of Laboratory Animals, as well as the European Community Council Directive of November 2010 regarding the treatment and use of laboratory animals (Directive, 2010/63/EU), which is equivalent to IACUC approval. The experiments involved male Wistar rats (OMD, Lublin, Poland) weighing 100–135 g at the beginning of the study. The rats were housed in pairs in cages measuring 55 cm × 33 cm × 20 cm, with a layer of sawdust about 3 cm thick on the floor. They had free access to both rodent chow (Sniff Spezialdiäten GmbH, Soest, Germany) and water throughout the study. The experiments were conducted under standard laboratory conditions, with a temperature of 22 ± 1 °C and a 12 h light/dark cycle (lights on at 7:00). Behavioral experiments took place between 8:00 am and 7:00 p.m. Prior to these experiments, the rats were handled for 5 min daily for 5 consecutive days. Two cohorts of animals were used in this study. Each group consisted of 7–10 animals, with a total of 125 animals included in the study (72 in the NOR group and 53 in the BM group).

### 4.2. Drugs

Ethanol (95% *w*/*v*, Polmos, Poznan, Poland) was diluted with saline (0.9% NaCl) to a 50% (*w*/*v*) concentration and administered intragastrically (i.g.) as a “binge-like” exposure at a dose of 5.0 g/kg, once daily for 5 days, based on a previously established method [40]. The control group of rats received saline instead of ethanol. All animals were gavaged with either ethanol or saline at the same time each day. The ethanol dose of 5 g/kg administered, i.g. for five consecutive days was selected to achieve high levels of intoxication, corresponding to blood alcohol concentrations observed in humans with heavy alcohol consumption. This approach allowed us to reliably model the effects of chronic ethanol exposure. Additionally, it is important to note that rats metabolize ethanol faster than humans [63], leading to a higher tolerance for elevated doses. The chosen dose ensured a consistent and reproducible impact on the target pathways while reflecting the physiological conditions associated with severe alcohol exposure in humans [36].

Org24598 lithium salt N-Methyl-N-[(3R)-3-phenyl-3-[4-(trifluoromethyl)phenoxy] propyl]-glycine lithium salt) was obtained from Tocris Bioscience (Bristol, UK) and administered intraperitoneally (i.p.) at doses of 0.1, 0.3, and 0.6 mg/kg. L-701,324 (7-chloro-4-hydroxy-3-(3-phenoxy) phenyl-2-(1H)-quinolone) was purchased from Merck, Sharp, and Dohme (Natick, MA, USA) and given at a dose of 5 mg/kg. All substances were dissolved in saline with 0.1% DMSO and administered in a volume of 2 mL/kg (i.p.). The doses of Org24598 [26,64] and L-701,324 [26,65] were selected based on previously published studies. In previous studies, L-701,324 at a dose of 5 mg/kg, had no effect on the behavior of control animals in memory tests but reversed the effects of Org24598 [26]. Org24598 was given 30 min before the testing session, while L-701,324 was administered 30 min prior to the Org24598 injection.

### 4.3. Research Outline

**Figure 7:**
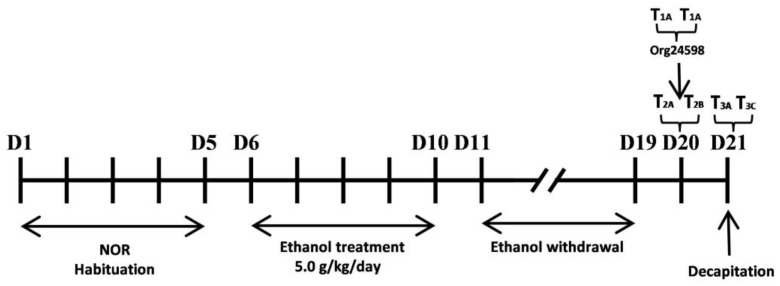
Diagram of the experimental design. The effect of withdrawal from binge-like ethanol administration in the novel object recognition (NOR) task performance. Effect of Org24598 given before first testing session on short (4 h) and long-term memory (24 h). D-day, T1 (A, A) (familiarization), T2 (A, B) (short-term memory), T3 (A, C) (long-term memory).

**Figure 8:**
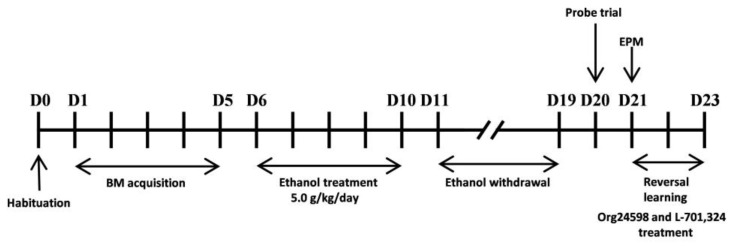
Diagram of the experimental design. The effect of withdrawal from binge-like ethanol administration in the Barnes maze (BM) task performance. Effect of Org24598 and L-701,324, given before every first reversal learning, on the cognitive flexibility impairment in the BM task and elevated plus maze (EPM). D—day.

At the beginning of the experiment, the animals were divided into two cohorts. One cohort (Experiment 1) was used to study the effect of Org24598 on ethanol withdrawal-induced memory impairments in the novel object recognition task (Figure 7). Following the test, the animals were decapitated, and their brains (PRC and HIP) were collected to examine the levels of the NMDA receptor subunits GluN1, GluN2A, and GluN2B, as well as the PSD-95 protein. The second cohort (Experiment 2) was used to investigate the effect of Org24598 on cognitive flexibility in the reversal learning task of the Barnes maze (Figure 8) in animals during ethanol abstinence. To examine the involvement of the glycine site of the NMDA receptor in the action of Org24598 in this experiment, the receptor antagonist L-701,324 was used.

### 4.4. Novel Object Recognition Task

The novel object recognition (NOR) task was conducted as previously described [35,66,67]. This task leverages the natural curiosity of rodents to explore new objects and is used to evaluate short- and long-term recognition memory, learning, attention, and related cognitive disorders in rodents. The test took place in a square plexiglass open box (63 cm long × 44.5 cm high × 44 cm wide) in a dimly lit room (30 ± 5 lx). Each rat underwent two 5 min sessions: a training session and a testing session.

Rats were habituated to the NOR apparatus over five days. During the training session (T1A, familiarization phase), they explored two identical objects (A1 and A2). After a 4 h interval, during the testing session (short-term memory), the rats explored one familiar object (T2A) and one novel object (T2B), differing in shape. Objects made of glass, plastic, and wood were selected based on preliminary experiments, confirming equal preference, and they varied in shape, color, and texture. Exploration was defined as sniffing or touching the object with the nose at a distance of less than 1 cm; sitting on the object was excluded. After 24 h (long-term memory), rats underwent a second test with one familiar (T3A) and one novel object (T3C), exploring freely for 5 min [40,67,68].

The placement of objects was counterbalanced within each group to prevent bias related to object or position preference. The box and objects were cleaned with a 10% ethanol solution between trials to remove any odor traces. A discrimination index (DI) was calculated to measure the ability to differentiate between the novel and familiar objects: (time spent with the novel object/(time spent with the novel + familiar object) × 100%). An index greater than 50% indicates a preference for the novel object, less than 50% indicates a preference for the familiar object, and 50% suggests no preference [69].

### 4.5. Barnes Maze Task

The Barnes maze (BM) task was conducted following the method previously described by Marszalek-Grabska et al. [40], with slight modifications. The apparatus (Stoelting, Dublin, Ireland) featured a circular gray metal platform (122 cm in diameter), elevated 100 cm above the ground, with 20 holes (10 cm in diameter) positioned around its perimeter. One of these holes was connected to an escape box (35 cm × 12 cm × 12 cm), made of the same material and color as the platform. The remaining holes were covered underneath with flat boxes of identical material and color, so the rats were unable to distinguish the escape hole from the others until they were directly beside it. Additionally, large, colorful geometric shapes were placed on the walls of the testing room, 1–2 m from the maze, to serve as visual cues. To stimulate an aversive escape response, the platform was brightly lit with two 500-watt lights positioned 1.5 m above the maze.

The Barnes maze task consisted of four phases: habituation, acquisition, probe trial, and reversal learning.

#### 4.5.1. Habituation Phase

The rats were habituated to the platform and the escape box (day 0) to reduce anxiety. This trial was performed with the lights on.

#### 4.5.2. Acquisition Phase

Twenty-four hours after habituation, the acquisition phase began. Over five consecutive days, the rats completed one training session per day. Each session consisted of two trials, each lasting 180 s, with a 5 min inter-trial interval during which the rats were returned to their home cages. The location of the escape hole remained constant throughout the acquisition phase. Each trial started by placing the rat at the center of the platform, allowing it to freely explore. The trial ended after 180 s or when the rat entered the escape box. Once inside the escape box, the hole was covered for 30 s before the rat was returned to its home cage. If the rat failed to locate the escape box within 180 s, it was gently guided to it by the experimenter and allowed to explore it for 30 s.

To eliminate odor cues and maintain consistency, the platform and escape box were wiped with a 10% ethanol solution after each trial. A trained observer recorded all trials. Since rats sometimes lacked motivation to enter the escape box even after locating it, as observed in previous studies [70,71,72], primary latency and primary errors were used as key measures. Primary latency was defined as the time it took for the rat to first make contact with the escape box, while primary errors were defined as the number of incorrect holes visited before the first contact with the escape box.

#### 4.5.3. Probe Trial

On the tenth day of ethanol withdrawal (day 20), the rats underwent a 90 s probe trial to assess their spatial memory. During this trial, the tunnel leading to the escape box was blocked [71,72], and the rats were allowed to explore the maze and investigate the escape box and the surrounding holes. Primary latency and primary errors in reaching the escape box were recorded.

#### 4.5.4. Reversal Learning Phase

One day after the probe trial (day 11), the rats underwent reversal learning trials for three consecutive days, with two 180 s trials conducted each day. These trials were like the acquisition trials, but the position of the escape hole was rotated 180°. This required the rats to relearn the escape location, as they could no longer rely on previously learned spatial cues. Before the first reversal learning trial, the rats were divided into experimental groups. Org24598 was administered once daily, 30 min before the first trial of each day for three days during the reversal learning session (control and ethanol groups). Data collected from these trials were used to calculate primary latency and primary errors. L-701,324 was administered 30 min before the Org24598 injection to one of the EtOH-treated groups.

### 4.6. Elevated Plus Maze (EPM) Test for Rats

The EPM apparatus was made of wood and elevated 50 cm above the ground in a quiet laboratory environment. It consisted of two open arms (50 cm × 10 cm) and two closed arms (50 cm × 10 cm × 40 cm). The floor-level illumination was approximately 100 lx. The test began by placing the rat at the center of the plus-maze, facing an open arm. The number of entries into each arm and the time spent in each arm were recorded for 5 min. An “arm entry” was recorded when the rat entered an arm with all four paws. The maze was thoroughly cleaned with a 10% ethanol solution between tests.

Anxiety-like behavior was evaluated using the following measures: (a) time spent in the open arms as a percentage of the total time spent in both open and closed arms, (b) number of entries into the open arms as a percentage of the total number of entries into both open and closed arms, (c) locomotor activity, assessed by the total number of entries into the closed arms [73].

### 4.7. ELISA Assay

Quantitative measurement of the GluN1 subunit in the rat brain structures was performed using a Rat Glutamate [NMDA] receptor subunit zeta-1 ELISA Kit (E1005Ra; Bioassay Technology Laboratory, Shanghai, China) following the manufacturer’s protocol. Firstly, frozen rat brain structures were homogenized in ice-cold PBS pH 7.4 containing cocktails of protease and phosphatase inhibitors (Sigma-Aldrich, Burlington, MA, USA) using a homogenizer ball (Bioprep-24, Aosheng, Hangzhou, China) (10 s at 10,000 rpm). Then, homogenates were centrifuged for 5 min at 5000 × *g* and the supernates were immediately removed. Duplicates of each sample and series of standards were transferred to ELISA plates. The absorbance was measured at a wavelength of λ = 450 nm using a Multiskan Spectrum spectrophotometer (Thermo LabSystems, Philadelphia, PA, USA). The concentration of protein was calculated from a standard curve and correlated with the amount of protein (ng/mg of protein). For protein measurement, the bicinchoninic acid assay (BCA) protein assay kit (Serva, Heidelberg, Germany) was used. All data were expressed as a % of the control.

The concentration of protein was calculated from a standard curve and correlated with the amount of protein (ng/mg of protein). For protein measurement, the bicinchoninic acid assay (BCA) protein assay kit (Serva, Heidelberg, Germany) was used. All data were expressed as a % of the control. Using an ELISA assay, the levels of NMDA receptor subunits were measured in brain structures, including the PRC and HIP, exclusively in animals that completed the NOR task, but not the Barnes Maze. In the HIP, measurements were conducted for the entire structure without subdivision into specific subregions. Following extraction, the brain tissues (PRC and HIP) were immediately frozen in liquid nitrogen to ensure the preservation of their integrity.

### 4.8. Experiment 1

After 5 days of the familiarization phase of the NOR task, rats (Cohort 1) were subjected to ethanol administration for 5 consecutive days according to a previously described method [35,36]. Next, on the tenth day of ethanol withdrawal (the 20th day of the experiment), Org24598 was administered at doses of 0.1, 0.3, or 0.6 mg/kg (i.p.), 30 min before the first testing session. To assess long-term memory, the second session of the NOR task took place 24 h after the first Org24598 administration (Org24598 was not given on this day) using the same animals.

### 4.9. Experiment 2

After the habituation and acquisition phases of the Barnes maze, rats (Cohort 2) were subjected to ethanol administration for 5 consecutive days. On the tenth day of ethanol withdrawal (the 20th day of the experiment), the probe trial was performed, and 24 h later, Org24598 was administered at a dose of 0.3 mg/kg, 30 min before each reversal learning session for three consecutive days. L-701,324 at the dose of 5mg/kg (i.p.) was administered 30 min before Org24598 administration to assess the involvement of the NMDA receptor glycine site in the effects of Org24598.

In the NOR/BM task, the following groups of animals were used: 0.9% NaCl (i.g)/Vehicle (i.p.), 0.9% NaCl (i.g)/Org24598 (0.1, 0.3 and 0.6 mg/kg, i.p.), ethanol (5g/kg, i.g.)/Vehicle (i.p.), and ethanol (i.g.)/Org24598 (0.1, 0.3 and 0.6 mg/kg, i.p.). In the BM task, additional groups, such as 0.9% NaCl/ethanol (i.g), L-701,324 (5 mg/kg, i.p.), and Org24598 (0.3 mg/kg) were used.

### 4.10. Statistical Analysis

The collected data were analyzed using Prism version 8.0.0 for Windows (GraphPad 8.4.3, San Diego, CA, USA). To evaluate the statistical significance of the effects seen in both behavioral and molecular tests, two- or three-way ANOVA with repeated measures was applied, followed by Bonferroni’s post hoc test. To evaluate BM probe trial results, unpaired Student’s t-test was used. The results are presented as means ± standard errors of the mean (SEM), with a *p*-value of less than 0.05 considered statistically significant across all tests.

## Data Availability

Data are contained within the article.
